# Successful desensitization in a boy with severe cow’s milk allergy by a combination therapy using omalizumab and rush oral immunotherapy

**DOI:** 10.1186/s13223-015-0084-y

**Published:** 2015-05-28

**Authors:** Masaya Takahashi, Shoichiro Taniuchi, Kazuhiko Soejima, Yasuko Hatano, Sohsaku Yamanouchi, Kazunari Kaneko

**Affiliations:** Department of Pediatrics, Kansai Medical University, 2-5-1 Shin-machi, Hirakata-city, Osaka 573-1191 Japan

**Keywords:** Rush oral immunotherapy, Omalizumab, Food allergy, Cow’s milk

## Abstract

**Background:**

Rush oral immunotherapy (OIT) combined with omalizumab (OMB) has been reported to be an effective and safe treatment for severe milk allergies. However, no report has described long-term follow-up observations after OMB discontinuation. The purpose of this case report was to evaluate the safety and efficacy of rush OIT in combination with OMB during a long period of treatment.

**Case presentation:**

A 5-year-old boy presented with a past history of two severe episodes of anaphylaxis (at the age of 2 and 3 years) after consuming small amounts of cow’s milk (CM). Before the OIT, the total immunoglobulin E (IgE) level was 654 IU/mL, and specific-IgE (sIgE) levels for CM, casein, and β-lactoglobulin were 77.0 kUA/L, 86.2 kUA/L and 12.0 kUA/L, respectively. The skin prick test (SPT) for CM showed a wheal (diameter, 20 mm) and erythema (diameter, 50 mm). In the open food challenge, he reacted to a 0.2 mL ingestion of CM and presented with dyspnea and laryngospasms, and he was then administrated 150 mg OMB every 2 weeks for 8 weeks. In the 9th week, he was admitted to hospital for the rush phase of the OIT. Once he was able ingest a dose of 200 mL CM without having an adverse reaction, he was discharged and allowed to continue a daily dose of 200 mL CM at home. During this phase, the sIgE levels were elevated, but the end-point titration values from the SPT gradually decreased, and the SPT was negative after 1 year of OMB treatment.

Five months after discontinuation of OMB, the daily CM ingestion was ceased for a 2-week period, followed by an oral food challenge (OFC) that was negative. The patient experienced only five mild adverse events during the course of rush OIT, even after the discontinuation of OMB and his quality of life improved dramatically afterwards.

**Conclusions:**

The combination therapy of rush OIT and OMB successfully maintained desensitization to CM in a boy with severe allergies. We propose that a negative SPT may be useful to guide discontinuation of OMB in such patients.

## Background

Rush oral immunotherapy (OIT) with omalizumab (OMB) has been reported to be an effective treatment for severe milk allergy with relatively low reaction rates during the rush phase of the rush OIT compared to that with the conventional OIT protocol [1]. This protocol involved OMB administration for a durations of only 4 months, and there were no description of adverse events after OMB was discontinued. Begin [2] et al. reported that, allergic symptoms temporarily increase after OMB is discontinued during a rush OIT protocol to multiple foods when combined with OMB. We hypothesized that OMB administration for a long period may reduce the frequency of adverse allergic reactions after treatment is discontinued. We present the case of a boy with severe cow’s milk (CM) allergy who is successfully desensitized after 12 months of OMB administration combined with rush OIT.

## Case presentation

We encountered a 5-year-old boy who had two severe episodes of anaphylaxis (at the age of 2 and 3 years) after he had ingested small amounts of CM products. Both times, he experienced facial erythema, dyspnea, hypotension, and loss of consciousness, and recovered after two epinephrine injections. He also received a glucocorticoid inhaler and leukotriene receptor antagonist for mild asthma.

The patient was initially tested for sIgE against CM, casein, and β-lactoglobulin using the ImmunoCAP assay system (Thermo Fisher Scientific, Uppsala, Sweden), and also underwent a skin prick test (SPT) and oral food challenge (OFC) for CM. Before the rush OIT, the total IgE level was 654 IU/mL, and the sIgE levels for CM, casein, and β-lactoglobulin were 77.0, 86.2, and 12.0 kUA/L, respectively. The SPT showed a wheal (diameter, 20 mm) and erythema (diameter, 50 mm). During the OFC, he reacted to 0.2 mL of CM and presented with dyspnea and laryngospasms. Thereafter, after we received ethical clearance from the ethics committee of Kansai Medical University (No. 502) for treatment of OMB, the patient was given 150 mg (10 mg/kg) of OMB every 2 weeks for 8 weeks, according to the procedure of a previous study for asthma [3]. At the 9th week, he was admitted to hospital for the rush phase of the OIT according to our previously described procedure [4]. In the OFC, he presented laryngospasms and cough in reaction to 5 mL CM. Then, the initial CM dose was set as 0.5 mL. After 14 days, he was able to ingest 200 mL of CM with only one adverse reaction, focal flush, during this phase.

The patient continued to ingest a daily dose of 200 mL CM until there was no adverse reaction to this dose, after which the patient was discharged, but allowed to continue the daily dose of 200 mL CM. During this maintenance phase of rush OIT, 150 mg OMB was continued every 2 weeks. Subsequently, the sIgE levels increased, whereas the end-point titration values from the SPT decreased gradually. The SPT was negative when the patient was tested 1 year later (Table 1). At this point, the OMB administration was discontinued. Ingestion of CM was continued during the next 5 months, and then stopped for another 2 weeks. The patient underwent the OFC after this period, and the results were negative. Throughout the rush OIT, including the period after the discontinuation of OMB, the patient only experienced five adverse reactions with daily CM ingestion, each with a grade of < 2 according to Sampson's criteria of anaphylaxis [5]. His quality of life dramatically improved after the combined treatment with OMB and rush OIT; he can now consume cheese and other milk products (e.g., cheesecake, and ice cream), and is able to exercise after ingesting these food products.

**Table 1:** Results of the skin prick test, total IgE, and sIgE levels over the course of OMB administration

Period of the start of OMB (months)	0	2	3	5	7	10	12	16	21
SPT^a^	1:10^4^	1:10^3^	1:10^3^	1:10	1:10	_^b^	1:10^3^	1:10^3^	1:10^2^
Total IgE (IU/mL)	655	1232	1443	1379	1430	1110	1294	565	310
sIgE (kUA/L)	77.8	347	687	359	252	223	78	37	7.3

*OMB* omalizumab, *SPT* skin prick test

^a^Seven serial 10-fold dilutions of fresh cow’s milk were prepared in sterile saline immediately before SPT. Each dilution (1:10, 1:10^2^, 1:10^3^, 1:10^4^, 1:10^5^, 1:10^6^, 1:10^7^) was placed at 4 cm intervals on the volar surface of both forearms. The mean weal diameter was calculated and the highest threshold of SPT was defined as follows: the highest dilution inducing a mean weal diameter of 3+ mm was considered the observed end-point dilution

^b^Indicates a negative result

## Discussion

One year of OMB administration combined with rush OIT may be a useful and safe treatment for patients with severe CM allergy, even after OMB was discontinued. In this case, at 1 year after OMB discontinuation, the patient was able to drink 200 mL of CM without any allergic reaction, and had not experienced exercise-induced anaphylaxis after the ingestion of milk products. Therefore, desensitization to CM seems likely to be due not only because of OMB administration alone, but also because of the combined immunotherapy.

In general, the patients should be monitored for at least 2–5 weeks after the discontinuation of OIT in order to determine if the patient has attained sustained unresponsiveness to an offending food allergen [6]. In this case, the period of discontinuation was relatively short (2 weeks). However, after the challenge, the patient can freely ingest CM and milk products such as cheese, and ice cream without experiencing any allergic reactions. Additionally, he is able to exercise after the ingestion of such food products. Therefore, we suggest that he has attained sustained unresponsiveness to CM.

Although the precise mechanism of underlying OIT and OMB combination is currently unknown, it is considered that during OMB administration, T-helper 2 (Th2) cell clones, which react to CM allergens CM allergens, may be lost in the low level of functional IgE blocked by OMB by the mechanism of oral tolerance induction by a high-dose exposure of allergen, which is mediated by lymphocyte anergy (absence of costimulation or interactions between CD28 on T cells and CD80/86 on antigen-presenting cells.) [7]. However, the clones are not entirely lost in the short period of OMB administration. OMB is expected to be effective in desensitizing patients to allergens for the duration of its use; however because of the potentially high medical costs and the adverse effects associated with prolonged treatment, it is necessary to predict when OMB can be discontinued in combination with rush OIT.

The SPT provides an ‘*in vivo*’ procedure for measuring the reactivity of sIgE antibody-activated mast cells and basophils. A rapid increase in the number of mast cells and basophils at the skin level, determined by SPT, has been observed in immunotherapy for both aeroallergens [8] and food allergens [9, 10].

In the present case, the CM-related sIgE levels were elevated during OMB administration, and rapidly decreased after OMB discontinuation, and thus are not useful in predicting desensitization. OMB is a humanized immunoglobulin G (IgG) antibody, with a half-life of 21 days; on the other hand, the half life of IgE is only 2 days. After administration, OMB binds with the IgE antibody, and the levels of various sIgE levels including CM-related sIgE increase.

Thus, during OMB administration, sIgE levels do not reflect desensitization, indicating that the SPT is the only way to predict desensitization.

## Conclusions

Although the mechanism of the combination treatment of rush OIT and OMB is unclear, this therapy may be successful for long-term allergen desensitization in children with severe CM allergy. Furthermore, we propose that the SPT may useful to guide discontinuation of OMB in such patients.

## Consent

Written informed consent was obtained from the patient’s parent for publication of this case report. A copy of the written consent is available for review the Editor-in-Chief of this journal.
